# Cochlear Implant Challenges in Children with Ichthyosis: A Systematic Review

**DOI:** 10.3390/genes16020129

**Published:** 2025-01-23

**Authors:** Valeria Caragli, Laura Luppi, Nicole Carrie Tegmeyer, Elisabetta Genovese, Davide Soloperto

**Affiliations:** 1 Otorhinolaryngology-Head and Neck Surgery, Audiology Program, University of Modena and Reggio Emilia, 41125 Modena, Italy; laura.luppi87@gmail.com (L.L.); 326249@studenti.unimore.it (N.C.T.); 2 Audiology Program, Department of Maternal, Child and Adult Medical and Surgical Sciences, University of Modena and Reggio Emilia, 41100 Modena, Italy; elisabetta.genovese@unimore.it; 3 Unit of Otorhinolaryngology, Audiology Program, Department of Maternal, Child and Adult Medical and Surgical Sciences, University of Modena and Reggio Emilia, 41100 Modena, Italy; davide.soloperto@unimore.it

**Keywords:** ichthyosis, cochlear implant, GJB2 mutation

## Abstract

Background/Objectives: Ichthyosis refers to a group of genetic disorders characterized by extensive scaling of the skin. Syndromic ichthyosis, such as KID syndrome, is associated with mutations in connexin 26, resulting in a triad of keratosis, ichthyosis, and deafness. Cochlear implant (CI) is considered an effective rehabilitation option for severe hearing loss in these patients, though challenges related to skin complications may arise. This study aims to systematically review the existing literature to evaluate the effectiveness of CI in patients with ichthyosis, focusing on auditory and communicative abilities. Methods: A comprehensive literature search was conducted across PubMed, Scopus, and Web of Science databases according to the PRISMA statement. Studies were included based on the presence of genetically confirmed ichthyosis patients who underwent CI. Results: A total of 29 studies were identified, of which 11 met the inclusion criteria, encompassing 47 patients. Genetic analysis revealed GJB2 mutations in 40 patients, with a prevalence of the c.148G>A (D50N) mutation. All patients experienced sensorineural hearing loss, predominantly severe to profound. CI was performed in all patients, with significant improvements in speech discrimination and auditory thresholds (89.4%). Complications post-implant were noted in 78.6% of cases, primarily involving wound infections and dehiscence. Conclusions: Despite the potential for significant complications, the overall outcomes suggest that CI can markedly enhance the quality of life of subjects. Multidisciplinary approaches and careful surgical planning are crucial to managing these patients effectively. Future research should aim for larger sample sizes and extended follow-up periods to further understand CI outcomes in this population.

## 1. Introduction

Ichthyosis represents a group of genetically determined Mendelian disorders of cornification, which leads to an excessive accumulation of corneal tissue. Ichthyoses are skin disorders characterized by extensive scaling that affects the entire skin surface, particularly life-threatening congenital ichthyosis in infants, which can lead to severe skin inflammation and complications like hypothermia and dehydration [1]. The presence of hyperkeratosis and ichthyosis as clinical entities is associated with a homeostatic reparative mechanism, resulting from an aberrant epidermal barrier [2]. Among the prevalent forms of ichthyosis, ichthyosis vulgaris is noted, with an occurrence rate of approximately 1 in 100 individuals, while recessive X-linked ichthyosis is encountered with a prevalence of about 1 in 4000. In non-syndromic ichthyosis, the phenotypic manifestations of the underlying genetic anomaly are restricted solely to the integument, whereas in syndromic ichthyosis, the condition is part of a broader syndrome, as the genetic defect concurrently impacts other organ systems [1].

KID syndrome represents one such syndromic variant, characterized as a rare congenital disorder defined by a triad of clinical features: keratitis, ichthyosis, and deafness. This syndrome is attributed to a dominant mutation in the connexin 26 gene, which adversely affects the epidermis, corneal epithelium, and inner ear, suggesting an ectodermal defect [3]. Patients harboring this mutation typically exhibit both sensorineural hearing impairment and visual disturbances, primarily due to progressive ocular keratitis, which culminates in intricate communication challenges. While the majority of cases present with congenital hearing loss, several instances of progressive auditory decline have been documented [4]. The dermatological manifestations are generally evident at birth and persist over time. In children with ichthyosis resulting from mutations in the GJB2 gene, hearing loss is predominantly linked to the disruption of gap junctions and their pivotal role in cellular communication within the inner ear [3]. Hearing loss in individuals with KID syndrome is typically severe or profound, non-progressive sensorineural hearing loss; however, moderate to profound sensorineural hearing loss has also been reported, along with conductive hearing loss resulting from external otitis and otitis media. While the connection between ichthyosis and hearing loss is not fully understood, it is hypothesized that the same genetic mutations affecting skin could also impact the inner ear’s development and function, possibly due to similar pathways involved in cell differentiation and communication [3,4,5].

In terms of rehabilitation, conventional hearing aids may prove ineffective due to skin issues and the accumulation of debris in the external ear canal as well as due to the degree of hearing loss. Thus, cochlear implant (CI) appears to be the most effective option for addressing severe hearing loss in these patients, as confirmed by the literature. Such procedures can pose risks for these individuals due to skin complications that may lead to impaired wound healing and extrusion of the implant [5]. Furthermore, patients with KID syndrome may experience visual impairment secondary to progressive ocular keratitis, which typically does not manifest at birth but develops over time, resulting in complex communication disorders. This must be carefully considered when making decisions regarding CI.

Despite the known challenges associated with hearing rehabilitation in this patient population, there is a significant gap in understanding the effectiveness and long-term benefits of CI for individuals with ichthyosis.

The aim of the present study is to systematically review the existing literature to evaluate the outcomes of CI in patients with ichthyosis, specifically focusing on auditory thresholds and communicative abilities, including perceptive and speech skills.

## 2. Materials and Methods

A comprehensive literature search was performed using PubMed, Scopus, and Web of Science databases, following the PRISMA 2020 guidelines [6]. The search utilized the terms “ichthyosis” AND “cochlear implant” without any filters or time restrictions. The final search was completed on 28 November 2024.

**Figure 1:**
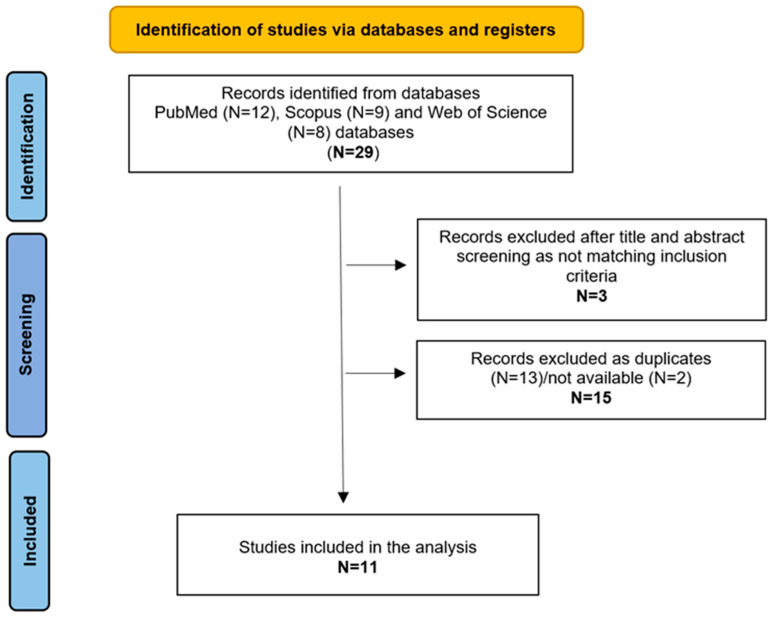
Identification process of the selected studies, according to PRISMA criteria.

The eligibility of the identified articles was evaluated based on three primary inclusion criteria: (1) studies that included patients with genetically confirmed ichthyosis; (2) studies involving patients with cochlear implants (CI); (3) studies that provided a clinical description of cases. Studies lacking clinical phenotype or genetic confirmation were excluded. Data from the selected studies were analyzed, extracting information on gender, age, genetic analysis, clinical features, imaging, audiology and language skills, as well as therapy and rehabilitation approaches. The retrieval process for selected articles is illustrated in Figure 1.

Data analysis was conducted according to the Helsinki Declaration, Italian privacy regulations, sensitive data laws, and the internal regulations of our hospital.

## 3. Results

A total of 29 studies were retrieved, of which 11 were included in the present study (Figure 1) [3,5,7,8,9,10,11,12,13,14,15]. Table 1 summarizes the data from the included reports.

### 3.1. Population

The patient population consisted of 47 subjects; among these, 8 were female, 3 were male, and the gender of 36 was unknown. The studies were conducted in different countries (Table 1).

### 3.2. Genetic Analysis

Genetic analysis was available for 40 of the 47 patients; even the 7 patients where the precise mutation was not clearly specified still had KID syndrome. The analysis identified a mutation in the GJB2 gene in all 40 patients. Twenty subjects had GJB2 mutations associated with deafness, while twenty had GJB2-unrelated deafness. The most common variation was a recurrent single nucleotide substitution, a c.148G>A (D50N) mutation [8,11,12,15], which was carried by five patients (25% of the cases with genetic analysis and mutations related to deafness). In the remaining cases, patients presented different variations of the mutation, such as c.149A>C (5%) [3], c.50C>T (5%) [10], and c.89T>A (5%) [12].

### 3.3. Clinical Features

Dermatological, ophthalmologic, and otologic (other than hearing loss) manifestations were the most common features reported among these patients (29.8%). In a study involving 32 patients [14], the clinical features had not been evaluated or reported, and in one study [8], one of the two patients did not have associated comorbidities. The most common dermatological manifestations included poor wound healing, alopecia, erythroderma/keratoderma, onychodystrophy, and dryness of the skin [3,5,7,9,10,11,12,13,15]. The most frequent ophthalmologic symptoms were photophobia, decreased visual acuity, corneal scarring, sensitivity to bright light, blepharitis, and neovascularization of the cornea [3,10,11,12,13]. Ear problems included keratitis of the external auditory canal and recurrent otitis media [3,5,13].

### 3.4. Imaging

Neuroimaging was reported in 5 patients out of a total of 47. It was performed using MRI (1 patient) [3] or CT scans (three patients) [3,8], while in two patients [10,11], the neuroimaging technique was not specified. Among these five patients, neuroimaging was negative in four out of five cases. The only patient with positive neuroimaging had undergone fetal MRI, which indicated hypoplasia of the cerebellar vermis and the fourth ventricle in continuity with a large posterior fossa midline. Postpartum MRI confirmed the findings of the fetal MRI, and a CT scan revealed a hypoplastic cochlea and a deficient root of the superior semicircular canal on the left side, as well as a small modiolus on the right side [3].

### 3.5. Audiological Evaluation

The audiological evaluation was reported in all 11 studies. Sensorineural hearing loss (SNHL) was diagnosed in 100% of the patients. The severity of hearing loss ranged from mild to profound, with most patients classified as having severe to profound SNHL (93.6%) [3,5,7,8,10,12,13,14]. One patient was diagnosed with mild to moderate SNHL at 9 months of age, which progressed to severe SNHL by the age of 2 years [15]. Two patients were diagnosed with SNHL without specific severity indicated [9,12]. Auditory brainstem response testing was conducted in five patients (10.6%), which did not detect wave V even with maximal stimulation [3,5,10,11]. Additionally, five patients underwent bilateral otoacoustic emissions, with no response observed [5,8,10].

### 3.6. Therapy, Auditory Rehabilitation, and Outcomes

In the 11 studies, it was specified that patients had received rehabilitation with hearing aids (HAs) before undergoing CI. Among these, 10 patients used bilateral HAs [3,5,7,8,9,11,12,13], while 1 patient used a single left HA [12]. All 47 patients evaluated in this review underwent CI; for 39 patients (83%), the side of the CI was not specified [3,7,8,9,12,14,15]. Three patients (6.4%) received bilateral CIs [12,13], three patients (6.4%) received right CIs [5,11], and two patients (4.3%) received left CIs [10,13]. Furthermore, the age at which 13 patients [3,5,8,9,11,12,13,15] underwent CI was reported, with an average age of 34.6 months (for patients with bilateral CIs, the age of the first implant was considered). For eight patients (17%), the Pure Tone Average (PTA) post-CI was specified, with a value of 37.5 dB across frequencies [3,5,7,8,10,11]. One patient reported an unsatisfactory result [9]. In the remaining 38 patients, only progress in auditory perception and language was reported [12,13,14,15]. A total of 89.4% reported and analyzed patients showed steady improvement in speech discrimination skills and speech production [3,5,7,8,12,13,14,15]. In the 2% (one patient) of patients with an unsatisfactory CI result, speech communication and comprehension skills were not achieved, and the patient relied almost exclusively on sign language and lip reading [9]. In 8.5% of cases, information on language and perception was not explored in depth [5,9,10,11].

### 3.7. Follow-Up and Complications

Data regarding the duration of follow-up post-cochlear implant surgery was available for only nine patients (19.1%) [3,5,8,12,13]. On average, patients were monitored and followed for rehabilitation for 45 months, with a minimum follow-up of 7 months [12] and a maximum of 10 years [8]. Complications were reported in 14 patients, while no complications were noted in 21.4% of these cases [5,12]. In the remaining 78.6%, the most frequent complications included wound infections and partial or total dehiscence of the surgical wound (60%) [3,7,8,9,10,12], otitis externa/media, and/or perforation of the tympanic membrane (30%) [8,13], and skin irritations with thickness reduction (20%) [11,13].

## 4. Discussion

It is well established that patients with GJB2 mutations, who also present with ichthyosis and hearing loss, may experience significant benefits from cochlear implant in terms of auditory perception, speech development, language acquisition, and communication skills [13]. Nevertheless, the management of sensorineural hearing loss in this patient population can be challenging due to the complex and frequently encountered complications associated with these conditions [13]. This study offers valuable insights into the feasibility of cochlear implant in patients with ichthyosis, juxtaposed with the critical issues surrounding this auditory rehabilitation solution.

In subjects with ichthyosis and hearing loss, the c.148G>A (D50N) mutation appears to be prevalent among patients with GJB2-related deafness, indicating a common pathogenic variant within the studied population. However, the presence of both GJB2 mutations associated with deafness and unrelated mutations highlight the intricate genetic factors contributing to hearing loss. These findings underscore the necessity for a comprehensive assessment of hearing loss severity to tailor appropriate interventions. In terms of auditory function, this review indicates that all patients with the condition exhibit sensorineural hearing loss, which significantly impacts communication skills. Specifically, a substantial majority of patients (93.6%) experience SNHL at a severe to profound level.

Therefore, a thorough evaluation of hearing loss severity is essential for developing suitable and individualized interventions. In addition to hearing loss, patients frequently present with dermatological and ophthalmological conditions, including poor wound healing and photophobia. Conversely, inner ear malformations are not commonly associated with this population. Nevertheless, notable findings in an individual patient suggest the potential for structural abnormalities linked to GJB2 mutations, also indicating that neuroimaging analysis could have implications for clinical management and patient prognosis.

To restore hearing function, hearing aids are typically recommended prior to cochlear implant, which aligns with standard practice aimed at maximizing auditory input prior to surgical intervention [16]. The outcomes of hearing aids are generally inferior to those of cochlear implants, primarily due to the degree of hearing loss. Furthermore, the effective utilization of hearing aids is often hindered by issues such as external ear canal obstruction or infection, as well as additional conductive hearing loss resulting from middle ear effusion or active otitis media with perforation [16].

Cochlear implant surgery is critical and represents the most effective rehabilitation solution for restoring auditory function [16]. A significant majority of patients undergo cochlear implant, indicating a pronounced trend toward surgical intervention within this population. The average age of implant ranges from as young as 9 months to as old as 25 years, emphasizing the critical role of early implant in optimizing speech and language outcomes. Consistent with existing literature, our study reveals that subjects with ichthyosis who underwent CI exhibited improvements in their average Pure Tone Audiometry (PTA) thresholds post-implant [13], as well as enhancements in auditory perception and language skill levels. Although some patients reported unsatisfactory outcomes—with one individual relying predominantly on sign language and lip reading—the overall trend indicates that CIs can significantly improve hearing capabilities in this unique cohort. The enhancement in auditory thresholds is vital, as it lays the groundwork for subsequent language acquisition and social integration, thereby improving the quality of life for these individuals. This is especially important for children, as early and effective communication skills are essential for academic and social success, as well as critical components of social interaction, which significantly influence emotional and psychological well-being [17].

However, complications following CI are common, occurring at a rate of 78.6%, and limiting the gradual acquisition of communication skills and quality of life. Consequently, the presence of complications in a significant proportion of patients indicates that while CI can be beneficial, it is not without its risks. Generally, major complications of CI surgery can include flap necrosis, improper electrode placement, and, though rare, facial nerve issues; minor complications may involve incision dehiscence, infections, facial nerve stimulation, dizziness, and issues with the Ineraid device pedestal [18]. Conversely, in patients with ichthyosis, the most frequent challenges post-implant are often related to skin integrity and the condition of the external ear canal. Specifically, wound infections and dehiscence, exacerbated by the underlying skin conditions, are the most common complications. Nonetheless, given the significant improvements in communicative abilities and the effectiveness of cochlear implants in enhancing patients’ quality of life, it is recommended to consider CI in this patient population. At the same time, the complex and frequent occurrence of complications suggests the need for careful planning of surgery and postoperative care, too. This approach includes intensive medical otologic management and close follow-up after surgery to facilitate implant success and address long-term issues [5].

On one hand, the age of the patient at the time of implant is essential for improving the outcome of the implant by leveraging synaptic plasticity. However, it does not appear that performing unilateral or bilateral CI, whether simultaneously or sequentially, affects the effectiveness of the implant or the complications related to the procedure.

Conversely, comprehensive preoperative counseling and a multidisciplinary approach involving dermatology and audiology specialists are critical to optimize outcomes and manage potential complications [3,4].

## 5. Conclusions

The study provides valuable insights into the genetic, clinical, and audiological characteristics of patients with GJB2 mutations and ichthyosis. It underscores the importance of comprehensive evaluations and highlights the potential for successful intervention through cochlear implant, while also recognizing the challenges associated with follow-up and complication management. Future studies may benefit from a larger sample sizes and longer follow-up durations to further elucidate the complexities of these cases.

## Figures and Tables

**Table 1:** Descriptive data of patients.

**Reference**	**N and Gender**	**Genetic Analysis**	**PTA**	**Imaging**	**Comorbidity** (except for common otological and skin problems connected to the disease itself)	**PA e CI (Age)**	**Outcome with CI: PTA and/or Language**	**Complications**	**Follow-Up**
Hampton et al., 1997 [7]	1 F	NR	Profound bilateral SNHL	NR	None	HAs, CI (unspecified whether mono-or bilateral)	Improved PTA Discrimination, speech and imitation improved steadily.	Infection and extensive wound dehiscence	NR
Smyth et al., 2012 [8]	2 (1M, 1F)	D50N: c.148G>A	Profound bilateral HL	CT neative	Shortening of Achilles tendon	HAs, Monolateral CI (for both patients)	Improved PTA and Speech. Poor discrimination, use of sign language	Otitis externa, progressive extensive wound dehiscence, CI explantation, intermittent ottorrhoea, TM perforation	10 y
Ahmadi et al., 2003 [9]	1 F	NR	SNHL	NR	Prematurity, carotenaemia, encephalomyelitis, shortening of Achilles tendon,	CI (9 m) (unspecified whether mono-or bilateral)	Unsuccessful CI	Poor wound healing	NR
Wang et al., 2023 [10]	1 F	D50N: c.50C>T p.S17F	Profound SNHL	Negative	Elderlylike appearance, photophobia	Monolateral CI	Improved PTA	Mild incision irritating, incision infection	NR
Choung et al., 2008 [11]	1 M	D50N: c.148G>A	Profound SNHL	Negative	Tongue with ulcerative and angular cheilitis, severely decreased visual acuity, neovascularization, corneal scarring, photophobia	HAs (1 y), Monolateral CI	Improved PTA	Difficulties in hearing rehabilitation due to low visual impairment, skin irritation	NR
Gumus et al., 2017 [5]	2 (1M, 1F)	NR	Severe -Profound SNHL	NR	Motor growth retardation	HAs (4 y;10 m), Monolateral CI (7 y; 5 y)	Improved PTA, perception and speech	None	1 y; 20 m
Arndt et al., 2010 [12]	2 F	D50N: c.89 T>A; GAC9AAC: p.Asp50Asn.	Severe progressive bilateral SNHL	NR	Skin manifestations, bright light sensitivity	- HA (16y), Sequential CI (20 y–25 y); - HAs (3 m), Sequential CI (14m-planned)	Improved open-speech comprehension awarness	Changes in skin morphology under the transmitter coil, skin necrosis, wound dehiscence and partial extrusion of the implant	7 m
Cushing et al., 2008 [3]	1 (gender unspecified)	D50A: c.149A>C	Profound bilateral SNHL	MRI: hypoplasia of the inferior cerebellar vermis and the fourth ventricle, large posterior fossa midline cyst; CT: hypoplastic cochlea, deficient roof of the SSC on the left and a small modiolus on the right.	Blepharitis, bilateral corneal pannus, mild vascularization, corneal	HAs (5 m), CI (1 y)	Improved PTA, perception and speech. Difficult in open set testing	Incision infection	4 y
Barker et al., 2009 [13]	3 (gender unspecified)	NR	Severe-Profound bilateral SNHL	NR	Gait problems, Shortening of Achilles tendon, calf and knee muscles, recurrent corneal infections, photophobia	HAs, - Sequential CIs (14 m, 47 m); - Monolateral CI (28 m); - Sequential CI (39 m, 62 m)	Improved speech and language	Abscess of the external ear canal and parotid, recurrent otitis media with otorrhoea, middle ear effusion, skin thickening over the site of the device, mastoiditis	42 m; 26 m; 12 m
Sinnathuray et al., 2004 [14]	32 (gender unspecified)	D50N: c.35G; D50N: c.35G/169C>T; deletion in GJB6; GJB2-unrelated deafness (20)	Profound SNHL	NR	NR	NR	Improved perception GJB2-related deafness> GJB2-unrelated deafness	NR	NR
Markova et al., 2016 [15]	1F	D50N	Severe bilateral SNHL	NR	None	HAs (30 m), Monolateral CI (42 m)	Improved PTA, perception speech and language	NR	7 y

Table’s 1 Legend: F: Female; M: Male; NR: Not Reported; SNHL: Sensorineural Hearing Loss; CT: Computerized Axial Tomography; MRI: Magnetic Resonance Imaging; HA: Hearing Aid; CI: Cochlear Implant; m: Months; y: Years; PTA: Pure Tone Average; TM: Tympanic Membrane.
